# Book Reviews

**Published:** 1891-09

**Authors:** 


TRANSACTIONS OF THE SOUTHERN SURGICAL AND GYNECOLOGICAL ASSOCIATION, Vol. III. Third session, held in Atlanta, Ga., November 11 and 13, 1890.

The Above Association has indeed performed a valuable service to the profession in issuing their third volume, and it is to the society's capable and efficient secretary, Dr. W. E. B. Davis, of Birmingham, Alabama, that credit is due for this valuable addition to our literature, showing, as it does, great care of preparation and a proper presentation of each subject, and we take great pleasure in commending the work.

---

THE WOMAN'S MEDICAL COLLEGE OF GEORGIA opens October 1st, with prospects of a large class. The ladies of the Board of Trustees, with Mrs. Governor Northen as President, and the Faculty, with Dr. A. G. Thomas as President and Dr. J. W. Stone as Dean, are bringing this institution to great usefulness to our Southern ladies. One-half reduction of lecture fees is granted to wives and daughters of physicians, clergymen and Confederate Veterans. For particulars, address Woman's Medical College, Atlanta, Ga., Box 215.

